# Learned features of antibody-antigen binding affinity

**DOI:** 10.3389/fmolb.2023.1112738

**Published:** 2023-02-21

**Authors:** Nathaniel L. Miller, Thomas Clark, Rahul Raman, Ram Sasisekharan

**Affiliations:** ^1^ Department of Biological Engineering, Massachusetts Institute of Technology, Cambridge, MA, United States; ^2^ Koch Institute of Integrative Cancer Research, Massachusetts Institute of Technology, Cambridge, MA, United States

**Keywords:** antibody, antigen, structure, affinity, features, classification, learning

## Abstract

Defining predictors of antigen-binding affinity of antibodies is valuable for engineering therapeutic antibodies with high binding affinity to their targets. However, this task is challenging owing to the huge diversity in the conformations of the complementarity determining regions of antibodies and the mode of engagement between antibody and antigen. In this study, we used the structural antibody database (SAbDab) to identify features that can discriminate high- and low-binding affinity across a 5-log scale. First, we abstracted features based on previously learned representations of protein-protein interactions to derive ‘complex’ feature sets, which include energetic, statistical, network-based, and machine-learned features. Second, we contrasted these complex feature sets with additional ‘simple’ feature sets based on counts of contacts between antibody and antigen. By investigating the predictive potential of 700 features contained in the eight complex and simple feature sets, we observed that simple feature sets perform comparably to complex feature sets in classification of binding affinity. Moreover, combining features from all eight feature-sets provided the best classification performance (median cross-validation AUROC and F1-score of 0.72). Of note, classification performance is substantially improved when several sources of data leakage (e.g., homologous antibodies) are not removed from the dataset, emphasizing a potential pitfall in this task. We additionally observe a classification performance plateau across diverse featurization approaches, highlighting the need for additional affinity-labeled antibody-antigen structural data. The findings from our present study set the stage for future studies aimed at multiple-log enhancement of antibody affinity through feature-guided engineering.

## 1 Introduction

Interactions between proteins are foundational in driving biological processes and critical for therapeutic interventions to treat diverse diseases. Two broad categories of protein-protein interactions include self-interactions to form oligomeric or tertiary protein assemblies and non-self-interactions between distinct proteins. The three-dimensional structure of proteins is critical for understanding and manipulating the molecular contacts between proteins in both self- and non-self-interactions. Therefore, the ability to reliably predict three dimensional structures of proteins is an important part of understanding the molecular interactions between proteins.

Protein structure prediction tools have continually evolved to improve reliability and accuracy in predicting three-dimensional structures of individual protein domains (AlQuraishi, 2021; Pearce and Zhang, 2021a; Pearce and Zhang, 2021b). Recently, the machine learning tool AlphaFold2 achieved an unprecedented leap forward in this effort (Wilson et al., 2022). This advance was followed by tools including AlphaFold-Multimer and RoseTTAFold that further demonstrated that the neural network deep learning methods can model protein complexes in addition to individual proteins (Baek et al., 2021; Evans et al., 2022). However, the success of obtaining reliable models of protein-protein complexes lags behind that of individual protein domains and is particularly challenging for antibody-antigen complexes (Akdel et al., 2022; Bryant et al., 2022; Yin et al., 2022).

Among the various non-self-protein-protein interactions, understanding and modeling the interactions between antibodies and antigens are of great interest from the standpoint of developing therapeutic antibodies (Norman et al., 2020). However, obtaining reliable models for antigen-antibody interactions has been challenging given that they involve non-self-interactions between distinct and highly flexible protein domains. While the three-dimensional structure of most of the regions of the variable Fab domain of an antibody can be modeled reliably (Leem et al., 2016), the loop region H3 of the heavy chain is often modeled with substantially lower confidence owing to a wide variety in its length and composition (Abanades et al., 2022; Ruffolo et al., 2022). Furthermore, predicting the right pose or mode of engagement of the Fab domain with the antigen is challenging given that several poses correspond to optimal molecular interactions (Fernández-Quintero et al., 2021).

Our early attempts to improve reliability of modeling antibody-antigen interactions around a decade ago involved using concepts from machine learning that are more widely employed currently (Tharakaraman et al., 2013; Robinson et al., 2015; Quinlan et al., 2017; Tharakaraman et al., 2018). At that time, we had featurized antibody antigen interfaces using various physicochemical and geometric properties of amino acids at these interfaces (Soundararajan et al., 2011; Quinlan et al., 2017; Tit-oon et al., 2020). Using these features, we developed various scoring functions and used linear regression methods based on these scores to reliably discriminate native poses of antibody-antigen complexes from decoy poses. Using this approach, we predicted a model of a broad-spectrum anti-Dengue virus antibody and engineered mutations that improved its affinity by more than 450-fold to Dengue virus serotype 4 while preserving and even moderately improving its affinity to the other three serotypes (Robinson et al., 2015). This was one of the early success stories for a computational method that was built using basic machine learning principles of featurization and regression to achieve such a drastic improvement in the binding affinity of an antibody (Wong et al., 2018). Of note, these physicochemical and geometric interface properties were likewise found to be useful for modeling viral escape from neutralizing antibodies, including instances where escape was conferred by combinations of epistatic mutations as occurred for the BA.1 Omicron variant of SARS-CoV-2 (Tharakaraman et al., 2014; Miller et al., 2021a; Miller et al., 2022a; Miller et al., 2022b).

Over the past decade, there has been a substantial increase in the available data on antibody sequences and antibody-antigen complex structures. Further, there have been significant advances in development of machine learning methods to build learning models from this information to simultaneously optimize multiple properties of antibodies including affinity and developability. The structural antibody database (SAbDab; https://opig.stats.ox.ac.uk/webapps/newsabdab/sabdab/) is a consistently annotated database of all antibody structures from the Protein Data Bank (PDB; http://www.rcsb.org/pdb/) (Berman et al., 2000; Dunbar et al., 2014). SAbDab also contains annotated information on the experimentally observed binding affinity for a structural antibody-antigen complex in the PDB (Schneider et al., 2022). Given that experimental binding data is associated with a three-dimensional structural complex and that the binding affinity values ranges over a five log scale, we postulated that we could employ learning methods to identify features in the antibody-antigen complex that are useful for discriminating strong and weak antibody-antigen binding affinity.

Importantly, this task is distinct from both our previous efforts to engineer antibodies toward higher affinity and recent efforts to develop machine learning models for prediction of antibody affinity (Myung et al., 2022; Yang et al., 2023). Specifically, these related works employ regression models trained on mutagenesis datasets, and as such perform best on antibodies with high homology to antibodies in the training set and spanning relatively narrow ranges of affinity from within two logs in the nM range, as the bulk of available structural data fall within this range. Instead, we chose to discriminate binding over a wider log-scale to facilitate approaches for rational engineering of antibodies for enhancing binding affinity over several log. Further, given the small dataset size (356 Ab-Ag complexes with numerous homologous Abs) it is critical that such features are derived using an approach that isolates the learning process from several sources of potential data leakage and erroneous signals.

We employed two distinct approaches to extract features that we postulated as useful for classifying high vs. low binding affinity. First, we considered features derived from previously learned representations of protein interactions, such as differentiable molecular surface interaction fingerprinting (dMaSIF)-site scores, significant interaction network (SIN) values, amino acid interface fitness (AIF) scores, and interaction descriptors annotated *via* PyRosetta including energetics, solvent exposed surface area (SASA), and surface complementarity (Chaudhury et al., 2010; Soundararajan et al., 2011; Tharakaraman et al., 2013; Gainza et al., 2019; Sverrisson et al., 2020; Lee et al., 2021). We refer to each of these sets of learned features as “complex feature-sets”. Second, we combined and contrasted these learned feature-sets with additional “simple feature-sets” computed directly from the Ab-Ag complexes. The simple feature-sets include features that measure the degree of complementarity determining regions (CDRs) involvement in the interaction, the number of amino acid contacts for the entire interaction (aa_counts) and for each complementarity determining region (aa_counts_by_CDR), and the multivalency of the interaction according to CDR involvement.

Herein, we investigate the ability of 700 features contained in the eight complex and simple features-sets to identify sub-nM Ab-Ag interactions. We observe that simple contact counting-based features perform as well or better than the complex feature-sets. Amongst the complex feature-sets, we find that network-based features perform better than energetics and prelearned features. Additionally, we demonstrate that a mix of features from all eight feature-sets provides the best sub-nM classification performance using the fewest total number of features (median cross-validation AUROC and F1-score of 0.72 *via* 16 features). The findings from the present study set the stage for future studies aimed at several-log enhancement of antibody target binding affinity in an “intelligent” fashion by focusing on improving features that show potential predictive value for identifying sub-nM Ab-Ag interactions.

## 2 Results

### 2.1 Dataset refinement and analysis

**FIGURE 1:**
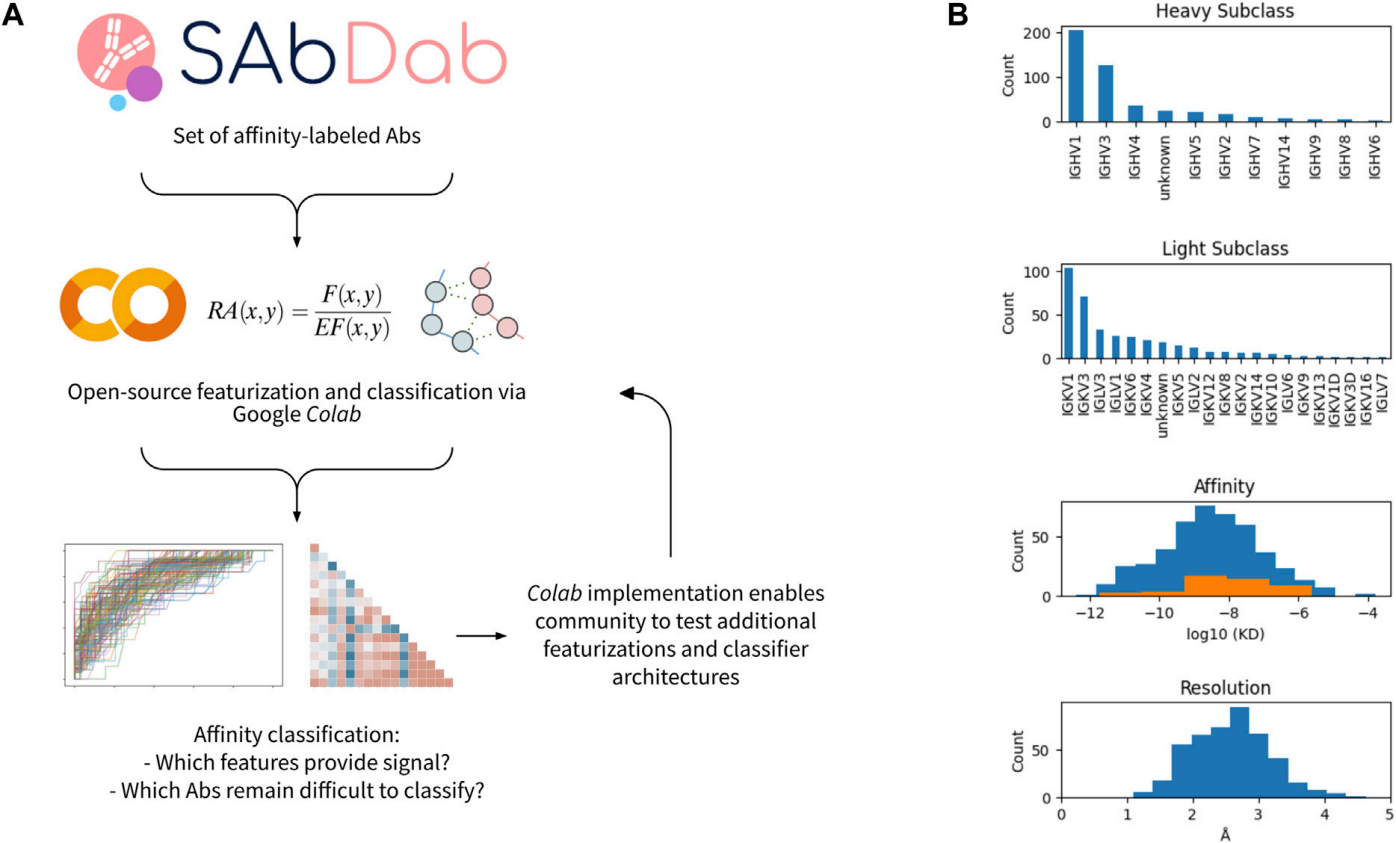
Workflow and data set overview. **(A)** Open-source workflow implemented via Google Colab. **(B)** Characteristics of affinity-labeled antibody data set from SAbDab. The counts of the antibodies displaying each heavy and light subclass are shown, as well as the distribution of antibody affinities and structural resolutions. For the affinity distribution, all antibodies included in the dataset are shown in blue, while the subset of antibodies containing a glycan or lipid in the epitope-paratope interface are shown in orange.

We performed a systematic investigation to benchmark distinct approaches and algorithms for featurizing antibody-antigen complexes. The workflow for the featurization and data analysis is shown in Figure 1A and implemented in an open-source Google Colaboratory (*Colab*) notebook. In brief, we first obtained a set of affinity-annotated antibody-antigen complexes from SAbDab. As described in the methods, we filtered this initial dataset to remove several sources of potential confounders and data leakage, including nanobodies and homologous antibodies. We subsequently pre-processed the remaining complexes, in which antibody sequences were renumbered using the Chothia scheme (Chothia and Lesk, 1987), heteroatoms were removed, missing side chains were replaced, and all complexes were relaxed *via* pareto-optimal relaxation (Nivón et al., 2013).

356 antibody-antigen complexes remained after filtering and pre-processing. The majority have heavy chain classes of IGHV1 or IGHV3, while the light chain subclasses were more diverse (Figure 1B). The median affinity in the dataset was 3nM, and the median resolution of the structures is ∼2.5Å. For both affinity and resolution, the dataset is approximately normally distributed (Figure 1B). However, the dataset is minorly skewed toward higher affinity and lower resolution, likely resulting from a selection bias in the choice of which antibodies are rigorously characterized and published. Of note, antibody-antigen interfaces containing non-protein components (e.g., glycans) comprise 13% of the dataset and are observed to have systematically lower affinity.

Subsequently, we applied existing featurization algorithms to every antibody-antigen complex in the dataset, as well *simple* descriptors of the interactions, to obtain 700 features describing each antibody-antigen interaction. Next, we evaluated the performance of all 700 features for their utility in classifying antibodies according to high- or low-affinity (1 nM cutoff). We employed a middle-dropped-out (MDO) dataset refinement, wherein all antibodies with affinity within one order-of-magnitude of the dataset median were removed to maximize the signal of features that best describe high- or low-affinity interactions spanning five logs of affinity. A classifier was selected instead of affinity regression (Myung et al., 2022; Yang et al., 2023) toward globally differentiating high- and low-affinity binders rather than predicting specific antibody ΔG values or ΔΔG values associated with point mutations.

### 2.2 Features relevant to classification of binding affinity

**FIGURE 2:**
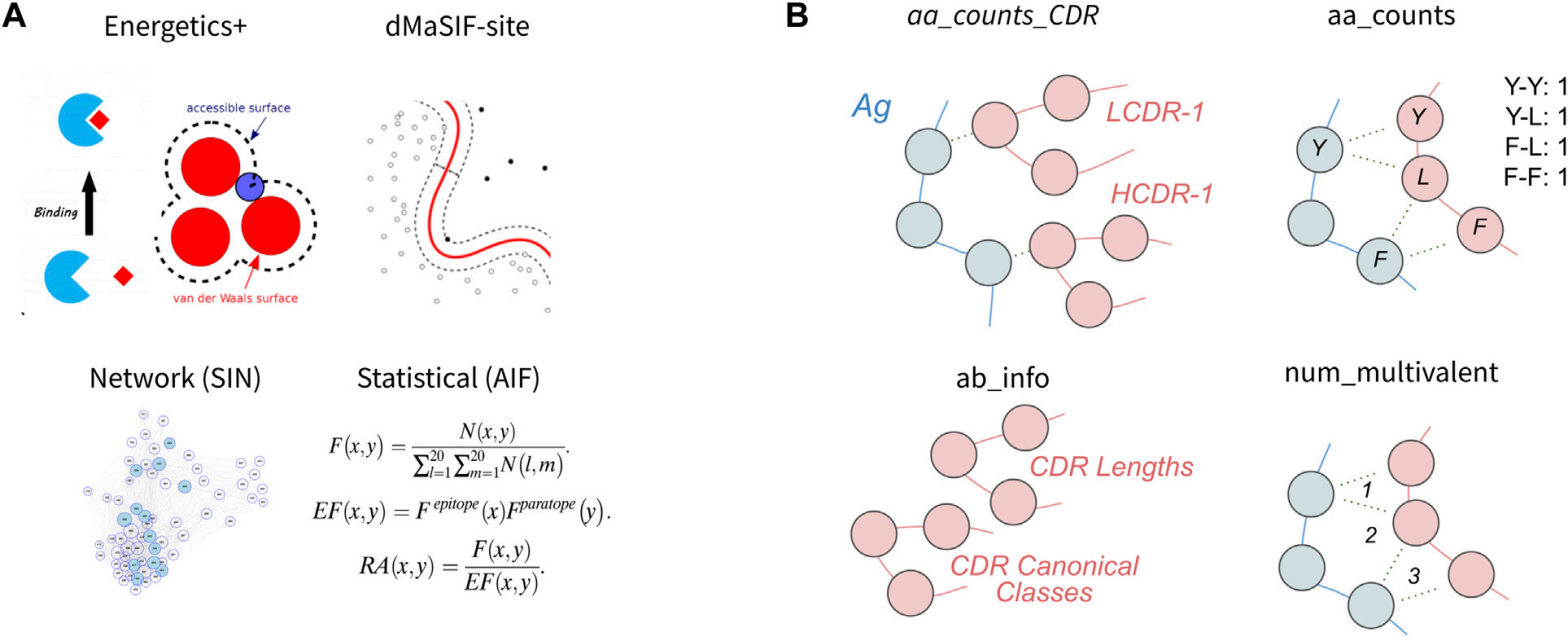
Features used to describe antibody-antigen interactions **(A)** Existing featurizations that have been validated for diverse protein-protein interaction modeling exercises. These featurizations model aspects of the antibody-antigen binding event such that they may be useful for affinity classification (see Methods). **(B)** Simple features that directly describe single components of the antibody-antigen interaction. These include the number of each combination of amino acid contact within the interface (aa_counts), the count of each interaction type for each CDR (aa_counts_CDRs), basic antibody descriptive information including the length and canonical class of each CDR (Ab_info), as well as the number of multivalent contacts spanning the epitope-paratope interface (num_multivalent_contacts).

In this section, we first evaluate four published featurization methods of protein-protein interfaces for classification of binding affinity (Figure 2A). Second, we evaluate four simpler featurizations that are directly computed from the antibody-antigen complexes (Figure 2B). For each feature set, we perform feature selection to obtain the 10 most discriminating features from the set and use these features to train a classifier. We then evaluate the cross-validation performance of the classifier on the task using the given feature-set. We also investigate the 10 features selected and their relative importance for the classifier, as well as correlative relationships amongst the 10 features. Finally, we aggregate features from the eight published and simple featurizations to obtain a ‘combined’ feature-set and evaluate the relative importances of the top features for the combined classifier.

#### 2.1.1 Performance of the existing feature-sets

We first investigate four existing featurizations: machine-learned (dMaSIF-site), energetics+ (PyRosetta), graph theory-based networks (SIN), and statistical descriptors (AIF) (Chaudhury et al., 2010; Soundararajan et al., 2011; Tharakaraman et al., 2013; Gainza et al., 2019; Sverrisson et al., 2020). For these four existing feature-sets, we obtain classifiers with median cross-validation AUCs and F-1 scores ranging from 0.58 to 0.69 and 0.67 to 0.70, respectively (Table 1). The network-based feature-set performed best (AUC = 0.69, F-1 = 0.70), while the statistical feature-set performed worst.

**TABLE 1:** Antibody affinity classifier ccross-validation performance across feature-sets. Summary of XGBoost (XGB) classifiers trained on each of the feature-sets, including feature numbers and classifier cross-validation (AUC and F-1 score). Performance of different classifier architectures, including RandomForest (RF), Support Vector Classifier (SVC), and Multi-layer Perceptron (MLP) are also shown for the "combined" feature-sets.

Feature-set	#Features in set (#used)	AUC/F1
Energetics	18 (10)	0.62/0.67
dMaSIF-site	26 (10)	0.62/0.67
Network (SIN)	26 (10)	0.69/0.70
Statistical (AIF)	26 (10)	0.58/0.67
aa_counts	400 (10)	0.61/0.64
aa_counts_CDR	150 (10)	0.67/0.67
num_multivalent	7 (7)	0.57/0.64
Ab_info	47 (10)	0.60/0.63
Combined-XGB	700 (16)	0.72/0.72
Combined-RF		0.70/0.73
Combined-SVC		0.66/0.74
Combined-MLP		0.67/0.71

**FIGURE 3:**
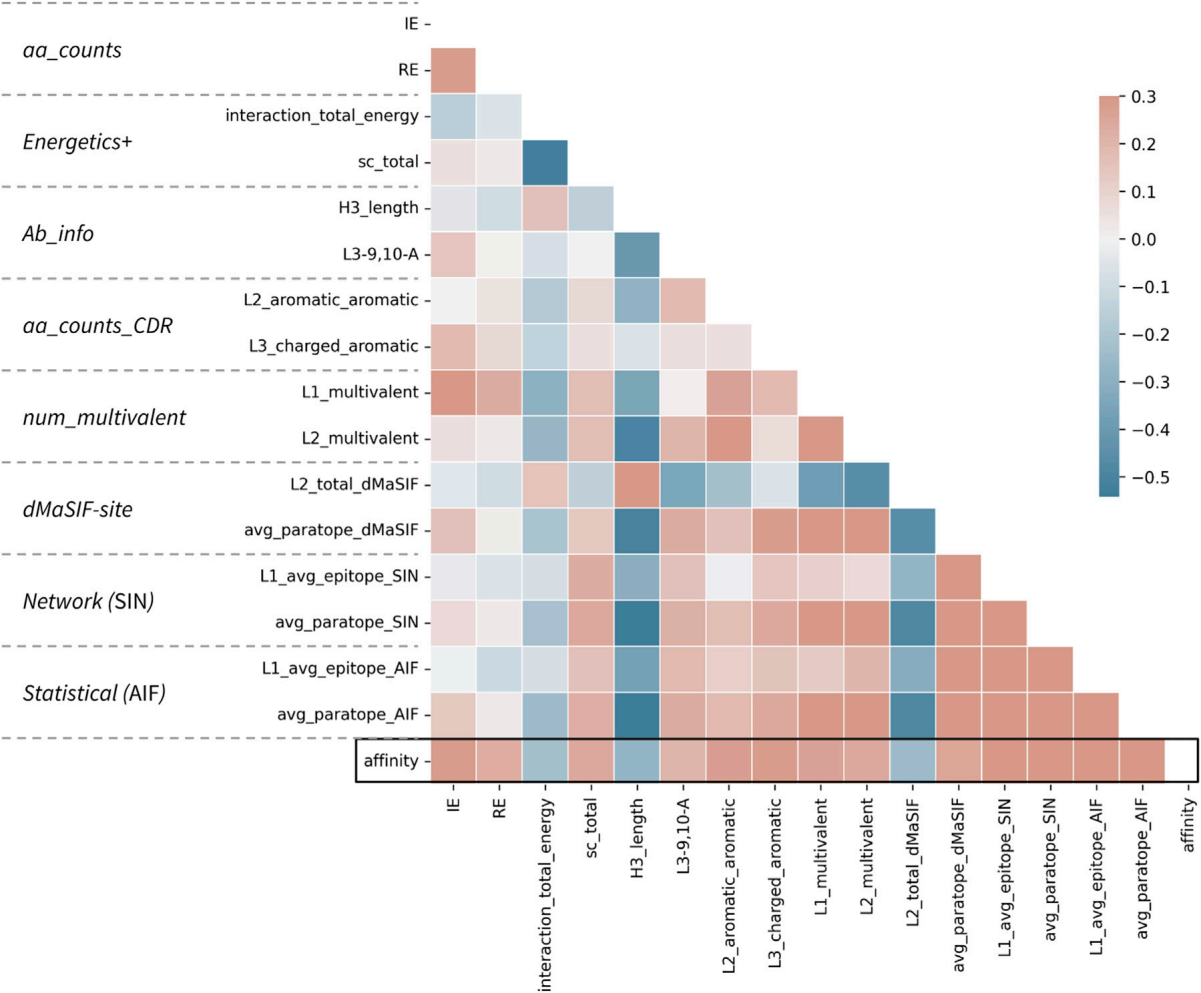
Correlations between top features and affinity for the combined classifier. Correlation matrix for the 16 features selected in the combined classifier versus each other and the affinity value. The parent feature-set for each feature is given at left. Affinity is treated as *−log10(KD [M])* such that a positive correlation (red) describes a feature (e.g., number of aromatic-aromatic contacts) that is positively correlated with higher affinity. Pearson correlation coefficients are shown according to the color scale and range from −0.5 to 0.3.

The top 10 features derived from the PyRosetta InterfaceAnalyzer and AntibodyDesign packages (referred to as the “Energetics” set) returned median AUC and F-1 of 0.62 and 0.67 (Table 1). The top two most important features in the feature-set are the total interaction energy for the interaction (interaction_total_energy) and the surface complementarity (sc_total) (Supplementary Figure S1). We observe these two features to be inversely correlated as expected (Figure 3). Features describing the isolated epitope energy score (epitope_total_energy), the interface energy (interface_dG), and the delta solvent accessible surface area upon antibody-antigen complexation (dSASA) are also of high importance. Amongst CDR-associated features, the CDR-H3 interaction energy ranks as most important, though CDR-features rank as less important than the aforementioned interaction-wide features.

The top 10 features derived from the dMaSIF-site predictions returned a median AUC and F-1 of 0.62 and 0.67 (Table 1). The two most important features within the dMaSIF feature-set are the average dMaSIF-site scores across the antigen epitope and the CDR-L1 (Supplementary Figure S2). dMaSIF-site scores for CDR-H3 and CDR-L2 also rank highly. In general, the dMaSIF-site features are positively correlated with each other and with affinity, though with the important exception of the light chain features describing total dMaSIF scoring of these CDRs. Additionally, across the dMaSIF-site features, dMaSIF-site scoring of the antigen ranks as more important than dMaSIF-site scoring of the antibody. This relationship is observed at the level of the entire interaction as well as for individual CDRs. This finding may reflect the fact that the dMaSIF-site training dataset predominantly included non-antibody protein-protein interactions, and further suggests that the standard dMaSIF training weights are more appropriate for scoring of epitope residues rather than paratope residues. Re-training a dMaSIF-site model on an antibody-enriched dataset may therefore improve the model’s capability to score paratope residues and to classify antibody affinity.

The top 10 features derived from the statistical (AIF) feature-set returned a median AUC and F-1of 0.58 and 0.67 (Table 1). The most important AIF feature is the average AIF across the entire epitope, followed by average epitope AIFs for the epitopes of the CDR-L1 and CDR-H3 (Supplementary Figure S1). As in the dMaSIF-site feature-set, the relative feature rankings display a trend toward feature descriptions of the epitope outweighing descriptions of the paratope. Further, average scores outrank total scores, suggesting that the average quality of the interaction is more useful than the total number of interactions weighted by quality. Interestingly, the top 10 AIF features are highly associated with each other, and these correlations are overwhelmingly positive in nature. This observation may result from the statistical definition of AIF scores, in which amino acid interface fitnesses are derived in aggregate across the epitope-paratope interface without specific information on CDR-location.

The top 10 features derived from the networking (SIN) features returned a median CV AUC and F-1 of 0.69 and 0.70—the top performing feature-set (Table 1). The top-ranking SIN features are consistent with the top three features for AIF—the average SIN for the L1-epitope, the entire epitope, and the H3-epitope (Supplementary Figure S4). Also consistent with the AIF feature-set, there is an enrichment of SIN features describing the epitope side of the interaction over the paratope side. Among the top 15 features, only four features reference the paratope—the average and total SIN for the L1, and the average SIN for the H3 and across the entire paratope.

#### 2.1.2 Performance of the simple feature-sets

Second, we investigated four simple featurizations that can be best defined as “counting” features. The four counting feature-sets are: *aa_counts* (the number of each combination of amino acid contact within the antibody-antigen interface), *aa_counts_CDR* (the number of contacts between each CDR and the epitope, split out by amino acid chemical type), *num_multivalent_contacts* (mulitvalent contacts within the interface), and *Ab_info* (summary information about each antibody such as CDR lengths and canonical class). For the counting feature-sets, we obtain classifiers with median cross-validation AUCs and F-1 scores ranging from 0.57 to 0.67 and 0.63 to 0.67, respectively (Table 1). The *aa_counts_CDR* feature-set performed best (AUC = 0.67, F-1 = 0.67), while the num_multivalent feature-set performed worst.

The aa_counts feature-set returned an AUC and F-1 of 0.61 and 0.64 when utilizing the 10 top features (Table 1). The most important features are enriched for interactions involving aromatic and/or charged residues, with eight of the top ten features including a charged/aromatic residue. Examining these top features in detail, it is clear that a number of AA interactions are enriched for either high- or low-affinity antibodies—but not both (Supplementary Figure S5). For example, alanine-serine interactions occur in numerous low-affinity but not high-affinity interfaces in the dataset. In contrast, Ab-glycine/Ag-Lysine interactions occur more frequently and in higher numbers per interface for high-affinity interactions. It will be valuable to observe if these trends are reinforced as additional affinity-labeled antibody-antigen structures are added to the SAbDab.

The aa_counts_CDR feature-set aggregates over the aa_counts features to summarize interaction types and CDR-locations (e.g., CDRL1-polar-charged). aa_counts_CDR returns an AUC of 0.67 and F-1 of 0.67 when utilizing the 10 top features (Table 1). The trend for top features is consistent with that of aa_counts, with all 10 of the top 10 features involving either an aromatic or a charged component. Interestingly, all five of the most important features describe light chain interactions, with four of these five describing CDR-L3 (Supplementary Figure S7). In contrast to aa_counts, wherein the top features identify interactions occurring uniquely for a small number of high- or low-affinity antibodies, the top aa_counts_CDR features appear to describe interactions in which the median counts for the entire distribution of high- and low-affinity antibodies are distinct (Supplementary Figure S6). This may explain the superior performance of aa_counts_CDR as compared to aa_counts, and additionally suggests it is a more robust feature-set than aa_counts.

The seven features measuring the multivalency of the interaction (*num_multivalent_contacts*) for each CDR and for the entire interaction returned an AUC of 0.57 and F1 of 0.64 (Table 1). The poor performance may occur because these seven features are highly correlated with one another (Supplementary Figure S7). This observation is easily explained, as the multivalency features share significant information content with each other—a multivalent interaction between L2 and L3 would be counted by both the L2 and L3 multivalency features. As such, the features return similar relative importance to each other, except for the feature describing the multivalency of the L2 interaction (L2_multivalent_count). The L2 multivalency feature ranks as more than twice as important as the next highest multivalency feature.

The 47 *Ab_info* features describe the length of each CDR and include a one-hot encoding of each CDR canonical class. The top 10 features in the Ab_info feature set returns an AUC of 0.60 and an F1 of 0.63 (Table 1). Examining the relative importance of the features indicates that H3 length is the most important feature by a large margin, followed by several features describing the canonical classes of light chain CDRs (Supplementary Figure S8). Interestingly, the lengths of each CDR are positively correlated with the antibody affinity (longer CDR implies higher affinity). The single exception to this trend occurs for the H3 length feature, wherein a longer H3 is associated with lower antibody affinity (Supplementary Figure S9). We discuss this finding in detail in the Discussion.

#### 2.1.3 Performance and complementarity of feature-set combinations

Finally, we selected a combined feature-set in which the top two features from each of the eight existing and simple feature-sets were aggregated. A classifier trained on the combined set achieves a CV AUC of 0.72 and F-1 score of 0.72 (Table 1). Of note, this represents the top cross-validation performance we report in this study when the dataset is carefully treated to remove sources of data leakage. When sources of data leakage such as homologous antibodies are not removed, cross-validation performance exceeds 0.80 (data not shown). Additional performance increases are also achieved when classifiers are not prevented from overfitting the small dataset; a cautionary note for future work in this area.

Examining the relative importance of all features derived from the combined feature-sets (Table 2) indicates that the most important feature describes the average amino acid networking of the epitope for CDR-L1 (L1_avg_epitope_SIN). The other top scoring features for the combined feature-set classifier rank at similar relative importance and include the number of isoleucine-lysine interactions, the number of charged-aromatic interactions on CDR-L3, the average paratope networking (SIN), the number of aromatic-aromatic interactions on the CDR-L2, and the interaction energy and surface complementarity.

**Table 2:** Combined classifier features and feature importances. Tabular summary of the most important features for the combined classifier. The importance of each feature averaged over 500 XGBoost trees is reported and ranked. The parent feature-set from which each feature belongs to is given.

Feature	Importance	Parent Feature-Set
L1_avg_epilope_SIN	0.154	*Network (SIN)*
IE	0.075	*aa_counts*
L3_charged_aromallc	0.075	*aa_counts_CDR*
avg_paratope_SIN	0.074	*Network (SIN)*
L2_aromatic_aromallc	0.065	*aa_ counts_ CDR*
lnteraction_total_onergy	0.064	*Energetics*
avg_paratope_dMaSIF	0.063	*dMaSIF-site*
sc_total	0.061	*Energetics*
RE	0.061	*aa_ counts*
L1_avg_epito po_AIF	0.055	*Statistical (AIF)*
avg_paratope_AIF	0.052	*Statistical {AIF)*
L2_total_dMaSIF	0.052	*dMaSIF-site*
L1_mullivalent	0.048	*num_mu/livalent*
H3_1ongth	0.040	*Ab_info*
L2_multivalenl	0.039	*num_multivalent*
L3·9.10·A	0.023	*Ab_info*

The top features spanning the eight feature-sets are on average less correlated than the top features within a given feature-set (Figure 3; Supplementary Figures S1–S8). This observation may explain the slight performance bump obtained when features from distinct sets are combined in this fashion. However, the top features, regardless of whether they derive from counting-, statistical-, energetics-, or network-based featurizations remain moderately associated, with absolute correlations up to Pearson r of 0.50 recorded. Most surprisingly, the strongest correlations are observed between H3 length and several network-based and dMaSIF features, suggesting that long H3s are associated with poor interaction quality as measured by distinct methodologies.

**FIGURE 4:**
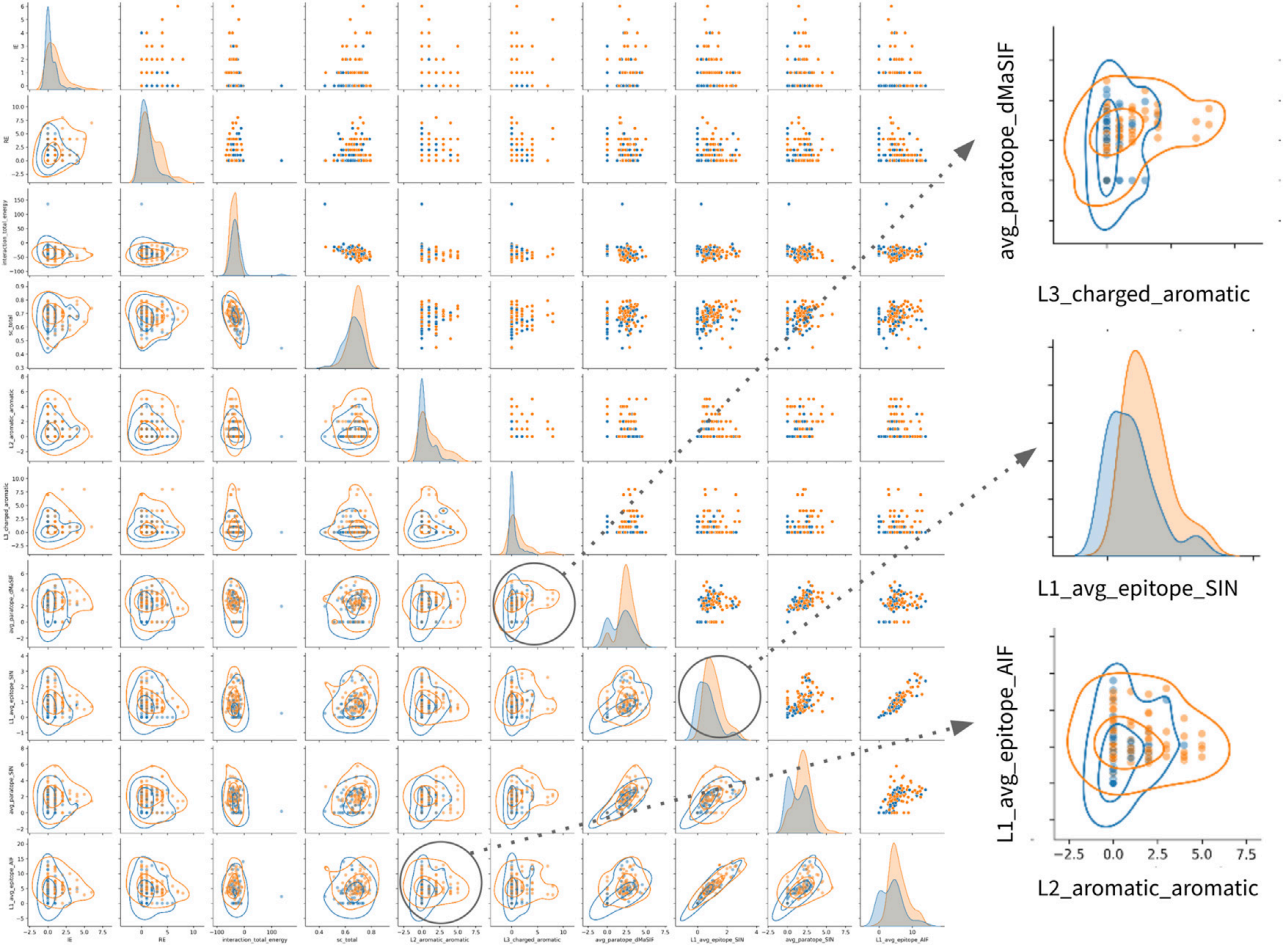
Relationships amongst most important features for classification of high- vs. low-affinity antibodies. Pairwise relationship matrix for the top 10 features across the feature-sets, as determined by feature importance rankings for th combined classifier (Table 2). For all plots, high- and low-affinity antibodies are shown in orange and blue, respectively. Cutoffs for high- and low-affinity are one-log above or below the dataset median affinity of 3nM (<300pM or >30nM). The raw data for each feature-pair are shown in the upper triangle. On the diagonal, kernel density estimates (KDE) for the distribution of each feature are shown. On the lower triangle, contour plots are superimposed on the raw pairwise data. Two pairwise associations and one KDE are enlarged on the right side of the figure. Additional pairwise relationship matrices for the top five features within each feature-set are provided in the supplement (Supplementary Figure S1–S8).

Finally, examination of the pairwise associations between features derived from distinct sets identifies several pairs that appear to offer complementary information for classification (Figure 4). The most striking pairs include L2_aromatic_aromatic + L1_average_epitope_AIF, and L3_charged_aromatic + avg_paratope_dMasSIF. The complementarity of these combinations suggests that high-affinity antibodies in the dataset utilize aromatic and charged interactions *via* one light chain CDR while not compromising the statistical quality (AIF) of the interactions for adjacent light chain CDRs.

Together, the most important features within each feature-set as well as within combinations of feature-sets appear to be enriched for features describing certain broad interaction qualities. These include: 1) contributions from charged and aromatic residues, especially when the charged/aromatic residues occur on the paratope (as historically observed (Zemlin et al., 2003; Birtalan et al., 2008; Robin et al., 2014)); 2) substantial participation of light chain CDRs with high quality interactions (as measured by statistical or network metrics); 3) favorable epitope character (as measured by dMaSIF, making direct interpretation of such character difficult); and 4) interaction networking, which appears best measured by network-based features rather than direct counts of multivalent network interactions, suggesting that higher-order network interactions rather than shallow networks are critical.

## 3 Discussion

In this study we used the affinity-annotated datasets of antibody-antigen structural complexes to identify features to classify affinity of antibody-antigen interactions. While the dataset in SAbDab is unique in terms of having simultaneous three-dimensional structure and annotated affinities for antibody-antigen complexes, the dataset is still small and therefore there is a performance limit for all of the classifiers. Due to the limited dataset size and our focus on understanding the information of feature-sets rather than in measuring absolute performance, we chose to compare the feature sets using a robust cross-validation process, where the dataset is randomized and the cross-validation is run fifty times to avoid biases introduced in AUC by small test sets as in Saal et al. (Saal et al., 2007). Thus, our method is more appropriate for determining the relative predictive power of various feature sets than if we tested all models on a small (<30 complexes) static hold out test set. In light of the small dataset size, we observe an upper limit of classifier performance despite implementing several distinct featurization approaches that span from first principles to statistical to machine-learned. Even when a large number of features exceeding the number of observations by a factor of five is utilized, the median AUC and F1-scores do not exceed ∼0.80. A similar performance increase is also observed when sources of data leakage are not properly removed from the dataset. Together, this suggests additional affinity-annotated structural data and novel featurizations may substantially improve the trained models.

Many of the antibody-antigen complexes include non-protein components in the interaction interface. These interactions primarily include anti-influenza hemagglutinin (HA) or anti-HIV envelope (env) antibodies that bind complex glycan-protein epitopes. Such antibodies often exhibit distinct features such as an unusually long CDR-H3 used to penetrate the immune-evasive glycan shields on these viruses (Miller et al., 2021b). A subset of structures in the dataset also includes acids or lipids in the interface. However, exclusion of antibodies with non-protein-protein interactions reduces classifier performance, even though none of the learned and basic feature-sets employed directly describe non-protein interactions. This finding suggests that the classifier can identify antibodies that target complex (e.g., protein + glycan) epitopes based on the conformation of the protein components of the antibody and antigen. Indeed, this may explain why individual features such as the CDR-H3 length may alone have such high classification value (Supplementary Figure S3). While this finding also suggests that the introduction of features describing non-protein-protein interactions could further improve top-line classifier performance, we chose not to pursue this route. Rather, we focus on the identification of features potentially useful for multiple log-fold affinity enhancement *via* protein engineering. As shown in Figure 1B, the dataset is essentially absent of very high-affinity antibodies that interact with glycan-epitopes. It is therefore unlikely that this dataset can provide meaningful insights into very high-affinity glycan- or lipid-based antibody interactions.

Our study adds to the recent body of work around machine learning predictors of antibody-antigen binding affinity by providing a comprehensive assessment of the information content of previously learned and basic counting features in a novel classification task. Recently, Myung et al. published CSM-Ab, which uses graph-based signatures to predict the binding affinity of antibody-antigen complexes, as well as rank docked poses (Myung et al., 2022). However, Myung et al. focus on regression rather than classification, and test their model on single and multi-point mutation data rather than on arbitrary antibody-antigen interactions. Such interactions are substantially more diverse. For example, the number of charged and aromatic CDR-H3 residues likely provides better regressive value for datasets that include a number of highly-homologous antibodies with identical light chains + CDR-H1/H2, whereas in this study all homologous antibodies were removed to maximize diveristy and minimize data leakage. Similarly, Yang et al. utilize a regression-based approach, employing area- and contact-based features to measure the predictive accuracy of numerous linear and non-linear models (Yang et al., 2023). Yang et al. show, amongst other findings, that Random Forests are superior to neural networks for this dataset size and featurization technique. Our resulting indicating superior performance of Random Forest and XGBoost as compared to a multi-layer perceptron support a similar conclusion. Further, our results suggest that counting and area-based features provide less value for global affinity classification as compared to affinity regression.

Complementing the aforementioned recent work, our approach focused on identifying features that perform relatively well at the related but distinct task of separating high- and low-antibodies when no labeled homology information is available (e.g., as would occur when evaluating a novel antibody). We also focus on benchmarking the performance of simple features such as amino acid contact numbers against higher complexity ‘learned’ features that have been previously developed. It is both useful and encouraging that we observe simple features to perform as well or better than learned features, as simple features bear fewer human biases and reduce computational complexity.

In line with previous reports in the literature (Zemlin et al., 2003; Birtalan et al., 2008; Robin et al., 2014), we find, employing a naïve approach, that features describing the number and chemical nature of CDR-H3 interactions are of high value for classifying antibody affinity. Across the feature-sets, H3-associated features rank highly. Also in agreement with generally-accepted concepts of antibody interactions, features describing charged and aromatic interactions are found to be of high value and enriched in the aa_counts and aa_counts_CDR feature-sets.

We make several unexpected observations as well. First, we find that a feature describing the length of the CDR-H3 ranks as more important than features describing the contact numbers or energy of CDR-H3 in a given interface. CDR-H3 length is found to be inversely correlated with binding affinity. We believe several factors may contribute to the inverse association observed between H3 length and affinity and the high importance of this feature: 1) antibodies with unusually long H3s often target heavily glycosylated epitopes (e.g., HIV *env*), as the long H3 is selected to accomodate glycans and glycan-engaging antibodies in our dataset tend to have lower affinity (Figure 1B); 2) there appears to be a tradeoff between H3 length and the number of CDRs that interact with the antigen, where the number of CDRs interacting with the antigen is correlated with higher affinity 3) there may be an elevated entropic cost associated with binding for longer H3s. We note that it is non-trivial to deconvolute the various causal vs. correlative contributions of these factors and others to the H3 length-affinity relationship, but highlight this relationship as a potentially fruitful avenue for future work.

Second, although H3 features are generally and broadly enriched, the most important features describing the quantity or quality of a given CDR interaction occur for the light chain. The top-ranking features that count aromatic and charged interactions do so for the CDR-L2 and -L3 rather than for H3. Moreover, the top statistical (AIF) and machine-learned (dMaSIF) features describing the quality of a specific CDR interaction also select light chain CDRs rather than H3. These findings support a conclusion that significant and high-quality contact for multiple light chain CDRs is more important for very high affinity interactions than a highly-optimized H3 interaction in isolation. Importantly, however, we note that the existence of numerous high-affinity nanobodies establish that light chaininteractions are not strictly required for very high affinity.

Third, the simple feature-sets we compute directly from the structural complexes perform nearly as well for classification as the complex network, statistical, and machine-learned feature-sets. This finding has several implications. Most importantly, for future work featurizing antibody-antigen interactions, simple features such as the top-performing aa_counts_CDR can be computed quickly and efficiently with minor performance loss. These features are less sensitive to structural resolution than more complex features such as energetics, and they do not require computationally-intensive and error-prone preprocessing workflows involving repacking and relaxation. For example, despite our efforts to implement an accurate yet computationally-reasonable energetics workflow, a single outlying structure recording implausibly high interface energy made it through to the classification stage (as can be seen in the raw data distribution for the interaction_total_energy feature in Figure 4).

Finally, we observe that network-based features (SIN) are the best performing individual feature-set. Historically, the SIN networking metric has been used to identify structurally-critical residues on viruses that mutate at lower rates due to this structural constraint. Observing a light chain SIN networking feature as the most important across all 700 features examined indicates that structural networking may also be useful in identifying and engineering high-affinity antibody interactions. However, there is room for improvement to better engineer these network features. For example, the SIN network implementation in this paper does not weight higher-order networks between CDRs any differently than those networks occurring within one CDR. This additional information may be useful to leverage in the future to develop better network features for this task.

The relative feature value within feature-sets and across combined feature-sets is interpretable because of the approach we have taken. Feature correlations are also annotated. These rankings and correlations inform future featurization and can also contribute to rational antibody design approaches—for example—via engineering multivalency and/or increased light chain involvement.

Several examples of outliers both in the category of low-affinity binders and high-affinity binders were observed in our analyses (summarized in Supplementary Figure S1). Through comparison of these outlying antibodies, we highlighted certain interaction features key to high-affinity binding events that appear to be poorly described by existing approaches. Specifically, it is observed that there is a blind-spot in the current approach for high-affinity antibodies that achieve this affinity without significant light-chain contributions. These analyses suggest fruitful avenues for future work to classify antibody affinity from complexed structure.

The open-source *Colab* notebook approach enables readers to quickly test their algorithms/features on the task at hand, and benchmark against existing approaches. It is our hope that our open-source implementation will therefore accelerate progress in antibody-antigen structural featurization and antibody affinity classification. In particular, we are excited to watch progress in this area, which we posit may stem most fruitfully from molecular dynamics simulations, geometric deep learning, and higher-complexity network algorithms. We also eagerly await expansion of the valuable affinity-annotated subset of SAbDab.

## 4 Methods

### 4.1 Google Collaboratory (*colab*) implementation and data/code availability

All code was implemented in a Google Collaboratory (*Colab*) notebook, and the code and raw data are further available on GitHub. We additionally provide the cleaned PDB files for each antibody-antigen complex. As certain features have a long runtime when applied across the entire dataset such that users using the free version of Colab would not be able to run the entire pipeline before timeout, we also host final feature-extracted files on the GitHub so that users of the free version can run the full pipeline while skipping certain feature extraction steps.

### 4.2 Antibody-antigen structural dataset acquisition and curation

The Structural Antibody Database (SAbDab) developed and maintained by the Oxford Protein Informatics Group [Dunbar et al., 2014] was accessed on 18 September 2022, and all antibodies in the database with labeled affinity and “protein” antigens were downloaded. Nanobodies were removed from the dataset as they were found to have systematically lower affinity than antibodies in the dataset (median affinity of 10 nM vs. 3 nM). Nanobody removal prevents the classifier from learning this format-affinity correlation which would exaggerate classifier performance. Further, several antibodies in the dataset are duplicates or derivates of one another. To prevent potential data leakage during cross-validation, we removed all antibodies with >95% heavy chain sequence identity, yielding a dataset of 356 affinity-labeled antibodies with a median affinity of 3 nM. For a given pair of high-identity antibodies, the antibody-antigen interaction with the affinity further from the dataset median affinity was selected. Additionally, for the classification task antibodies with affinities within one order of magnitude of the dataset median were removed. We refer to this set consisting of 142 antibodies as the middle-drop-out (MDO) set.

### 4.3 Antibody-antigen complex pre-processing

All antibody-antigen complexes were pre-processed prior to feature extraction and analysis. The antibody-antigen complex pre-processing comprised of: 1) sequence renumbering to the Chothia numbering scheme, 2) removal of water molecules and ligands, 3) replacement and repacking of missing side chain atoms in PyRosetta, 4) all complexes were relaxed in PyRosetta *via pareto-optimal* relaxation. Pareto-optimal relaxation minimally perturbs backbone structure (mean RMSD < 1 Å), provides optimized performance for protein design tasks (Nivón et al., 2013), and is utilized for antibody-specific tasks as in the RosettaAntibodyDesign framework (Adolf-Bryfogle et al., 2018). The pre- and post-relaxation processed antibody-antigen complex structures are available on the GitHub that accompanies this manuscript.

### 4.4 Feature extraction for external feature-sets

Features derived from existing published algorithms or workflows, which are referred to as “complex” feature-sets, were implemented and extracted as follows.

• ***dMaSIF-site***: dMaSIF site is a machine-learned annotation of protein surface residues in which each residue is scored according to the likelihood that the given residue participates in a protein-protein interface based on combined geometric and chemical features. All protein residues in each antibody-antigen complex were scored using the following dMaSIF-site model: 3Layer_16dims_epoch85; using the following parameters: model_resolution = 0.7 Å, patch_radius = 9 Å, subsampling = 150. From these residue-wise scores, 26 dMaSIF-site features were generated: the average and total dMaSIF-site score for each CDR (12 features) and each CDR-epitope (12 features), and the average dMaSIF-site for the entire epitope and paratope (2 features).
• **E*nergetics/PyRosetta***: 15 features generated *via* the PyRosetta InterfaceAnalyzerMover and RosettaAntibodyDesign applications were extracted. The 18 features extracted included: the number of epitope residues calculated using *select_epitope_residues()*; the epitope SASA calculated using *SasaMetric()*; the epitope total energy calculated using *TotalEnergyMetric()*; the interaction energy calculated using *InteractionEnergyMetric()*; the crossterm interface energy calculated using *get_crossterm_interface_energy()*; the complex delta free energy calculated *via get_interface_dG()*; the separated interface energy ratio calculated using *get_separated_interface_energy_ratio()*; the total complex energy calculated using *get_complex_energy()*; the antibody and antigen normalized scores calculated using *get_side1_score()* and *get_side2_score()*; the interface surface complementarity and dSASA according to the InterfaceAnalyzerMover variables *sc_value* and *dSASA*; and the interaction energies for all six CDRs individually calculated *via InteractionEnergyMetric().*
• ***Network*** (***SIN***): All antibody-antigen complexes were modeled as Significant Interaction Networks (SIN) as previously described (Soundararajan et al., 2011). Briefly, antibody-antigen complexes were converted to network representations in which each amino acid was defined as a node, and edges were constructed based on inter-residue interactions computed from a set of eight weighted interaction types. Subsequently, the edge weights denoting interactions across the epitope-paratope interface were summed for each epitope or paratope residue to obtain a single score describing the degree of networking between the given epitope/paratope residue and the cognate surface (e.g., networking between CDRH3 TYR101 and the entire epitope). 26 SIN features were generated from these residue-level scores as the average and total SIN-score for each CDR (12 features) and each CDR-epitope (12 features), and the average SIN for the entire epitope and paratope (2 features). The significant interaction networks for all antibody-antigen complexes are available at the GitHub accompanying this manuscript.
• ***Statistical*** (***AIF***): The Amino Acid Interface Fitness (AIF) was computed for every interface residue on each antibody-antigen complex as previously described (Tharakaraman et al., 2013). Briefly, every amino acid pairing across the epitope-paratope interface is scored based on the propensity for observing the given amino acid pairing in a dataset of 84 non-redundant antibody-antigen complexes. While there may be partial overlap between the antibody-antigen complexes the AIF weights were trained on and those employed in this paper, this is unlikely to introduce bias as the AIF scoring scheme did not include affinity annotation or affinity weighting. 26 AIF features were generated from the residue-level AIF scores, which include the average and total AIF for each CDR (12 features) and each CDR-epitope (12 features), and the average AIF for the entire epitope and paratope (2 features).

### 4.5 Feature extraction for simple feature-sets

Further, we extracted several relatively straightforward features computed directly from the antibody-antigen complexes, with all code implemented in the accompanying Google Colab notebook. These features were computed as follows.

• ***aa_counts***: Every pairwise amino acid interaction (e.g., Tyr-Tyr or Ser-Thr) within the antibody-antigen interface was counted resulting in 400 features (20 AA x 20 AA). Two residues were denoted as potentially interacting and thus were counted if the distance between their carbon alpha residues was less than the length of their combined sidechains plus an interaction distance of 4.5 Å, wherein the sidechain length was measured as the distance between the amino acid alpha carbon and the furthest side chain heavy atom in the pareto-optimal relaxed pose. Pre-processing scripts for these computations were adapted from: https://github.com/HeliXonProtein/binding-ddg-predictor (Shan et al., 2022) and https://github.com/dauparas/ProteinMPNN (Dauparas et al., 2022).
• ***aa_counts_by_CDR***: A second feature-set was generated from the aa_counts feature-set, in which the aa_counts feature-set was reduced to 150 features that abstracted the aa_counts by residue chemical type and assigned them to CDRs. In short, each amino acid was assigned a chemical type based on canonical definitions: charged = R, H, K, D, E; aromatic = F, Y, W; polar = S, T, N, Q; hydrophobic = A, V, I, L, M; special = C, G, P, as well as a location based on CDR. Every interaction type for each CDR was then counted. This resulted in 25 chemical interaction types (e.g., aromatic-aromatic or polar-charged) for each of the six CDRs, producing a total of 150 features (e.g., H3_aromatic-epitope_aromatic). Interaction cutoff distances were computed as for aa_counts.
• ***num_multivalent_contacts***: A feature-set describing the degree of multivalency of the interactions for each CDR as well as for the entire paratope was generated, resulting in seven features. First, for each residue on the antigen, the number of distinct CDRs the given antigen residue interacts with was computed according to the interaction distance cutoff used in the aa_counts calculation. Subsequently, for each CDR, the number of epitope sites the given CDR interacts with that also interact with two additional CDRs was summed. For example, the H3_multivalent feature would be assigned a value of four if the CDR-H3 interacted with four epitope residues that each interacted with at least two other CDRs. Finally, a seventh feature describing the multivalency of the entire interaction was computed as the number of CDRs with at least five multivalent interactions each.
• ***Ab_info***: A feature-set describing basic information about each antibody was generated with 57 features. The first six features describe the length of each CDR, and the subsequent 51 features represent a one-hot encoding of each CDR canonical class. CDR length and class were annotated using SCALOP (Wong et al., 2019). All CDRs with failed class assignment were aggregated under a single one-hot entry labeled “None.” The North CDR definition was used for defining all CDRs and calculating CDR lengths (North et al., 2011).

### 4.6 Affinity classifier implementation

We implemented a binary:logistic XGBoost classifier to classify antibodies according to greater than or less than 1 nM affinity using a variety of features and feature-sets (Chen and Guestrin, 2016). XGBoost hyperparameters were not tuned. For each classifier, 90/10 cross-validation was performed 50 times, where a new random split was generated for each of the 50 runs to minimize the noise introduced by the small validation-set. These 500 cross-validations were used to compute median area under the receiver operating curve (AUC) and F-1 scores. Features were selected using sklearn’s SelectKBest() and the f_classif selection metric (ANOVA F-value). For each feature-set classifier, the top 10 features within the set were selected and utilized by the classifier. For the ‘combined’ classifier, the top two features from each feature-set were selected and then aggregated to obtain a total of 16 features. To confirm feature-observation associations, affinity labels were randomized and classifiers trained on the randomized dataset were confirmed to return an AUC of 0.50 ± 0.01. Further, normalized feature importance for each feature-set were obtained *via* XGBoost’s *feature_importances_* attribute, which describes the mean gain for each feature across all splits. Several alternative classifier architectures including RandomForest, GradientBoosting, Support Vector (SVC), and Multi-layer Perceptron (MLP) were also implemented *via* sklearn and not tuned. In addition to classifier architecture, readers may adjust several variables in the Colab notebook including: 1) the affinity-cutoff for classification, 2) the number of cross-validations to perform, 3) the cross-validation training vs test set split, 4) the range of affinities to use for training and cross-validation, 5) the number of total features or features from each feature-set to utilize, and 6) whether to include nanobodies and/or high-homology antibodies in the experiment, which demonstrates the performance gain as a result of data leakage.

### 4.7 Identification of recurrently misclassified antibodies

The number of times that each antibody was misclassified by a classifier trained on each feature-set was tracked during the fifty-fold cross-validation process for each classifier. Antibodies that were incorrectly classified at rate of ≥80% for at least seven of the nine feature-sets were identified as recurrently misclassified antibodies for which classification performance was very poor across all investigated feature-sets. Recurrently misclassified antibodies were subsequently manually investigated through examination of 1) relative/marginal performance of feature-sets on these antibodies, 2) specific scoring of these antibodies *via* the most important features within feature-sets, 3) literature review of known structure-function relationships for these antibodies.

## Data Availability

The data and code that support this study are openly available in Github at https://github.com/nkmiller/protein_interactions.
