# Supplementary material for: Learned features of antibody-antigen binding affinity
Source: Front Mol Biosci. 2023 Feb 21;10:1112738. doi: 10.3389/fmolb.2023.1112738 (PMC9989197; doi:10.3389/fmolb.2023.1112738)

# *Supplementary Material*

# Predictive Features of Antibody-Antigen Binding Affinity

**Nathaniel L. Miller[1], Thomas Clark[1], Rahul Raman[1] and Ram Sasisekharan [1,*]**

*** Correspondence:** Ram Sasisekharan: rams@mit.edu

## 1 Examples of Antibodies that were outliers in the classification of binding affinity

Certain antibodies were outliners in the high or low affinity binding classification regardless of the feature-set employed. Some of these outliers that were consistently incorrectly classified by the majority or all of the nine classifiers (the seven individual feature-sets and the two combinations of feature-sets) were examined further.

### 1.1 High-affinity outliers:

- **1P2C**: F10.6.6 is an anti-lysozyme antibody that was affinity-matured three orders of magnitude from ~100nM to ~0.1nM. Seven of the nine classifiers, including the best performing classifiers and their combinations, regularly fail to classify F10.6.6 as a high-affinity antibody. The two classifiers that perform relatively better on F10.6.6 classification are those that employ the dMaSIF and num_multivalent_contacts feature-sets. This suggests that the high-affinity nature of the interaction may be at least in part encoded by 1) the high-scoring epitope (via dMaSIF-site) on lysozyme or 2) the significant multivalency of the interaction between F10.6.6 and lysozyme. However, other feature-sets employing other features (e.g., energetics or aa_counts) appear to perform quite poorly at recognizing the high affinity of the interaction.
- **5J13**: Tezepelumab or MEDI9929 / AMG-157 is an anti-cytokine (TLSP) antibody with an affinity of 1e-10.2. Seven of the nine classifiers, including the best performing classifiers and their combinations, regularly fail to classify Tezepelumab as a high-affinity antibody. The two classifiers that perform relatively better on Tezepelumab classification are those that employ the Ab_Info and Energetics feature-sets. As evidenced by the feature-importances for the top performing feature-sets and combinations, these classifiers place a very heavy importance on interaction-multivalency and the involvement of the light chain. Upon examination of 5J13, it is immediately evidence that the Tezepelumab light chain is relatively poorly involved in the interaction owing in part to the small cytokine antigen target. The top classifiers obtained in this manuscript may therefore have difficulty identifying sub-nanomolar antibody interactions that are based heavily on the heavy chain with little dependence on the light chain.

### 1.2 Low-affinity outliers:

- **3UYP**: 4E11 is a Dengue virus (DENV)-targeting antibody with variable affinity across serotypes. In structure 3UYP, 4E11 is bound to DENV serotype 4 with a very low affinity of 1e-

5.4. 3UYP is the only structure that all nine classifiers failed to classify correctly 100% of the time. Interestingly, three other structures of 4E11 bound to the other 3 serotypes of DENV are included in the dataset, all three of which are significantly higher affinity (from 10nM to 0.1nM). The implemented features which largely focus on aspects of the antibody rather than the antigen may therefore evaluate 4E11 as more high-affinity-like and miss nuances of the complex with DENV serotype 4 derived from the epitope-side of the interaction. Additionally, 4E11 is known to interact with a quaternary epitope on DENV, whereas the analyzed complex only includes 4E11 complexed to the DENV-4 envelope protein domain III, such that valuable tertiary and quaternary context is lost. This outlier therefore highlights that one avenue for future featurization includes features that are able to distinguish the affinity of cross- or broadly-neutralizing antibodies bound to different targets on the basis of the distinct antigens or the distinct interfaces formed with each antigen.

- **3C09**: Matuzumab, an EGFR-targeting Ab with affinity of 1e-7.4. Poorly classified by eight of the nine classifiers examined, with the exception of energetics. This is part of a trend where energetics/dMaSIF perform better for such interactions.
- **6B0S**: 1710, a Plasmodium falciparum sporozoite-targeting Ab with affinity of 1e-6.8. Poorly classified by seven of the nine classifiers examined, with the exception of energetics and dMaSIF. This is part of a trend where energetics/dMaSIF perform better for such interactions.

# 2 Supplementary Figures

## 2.1 **Figure S1:** Pairwise association matrices for the top 5 features within the PyRosetta (Energetics+) feature-set.

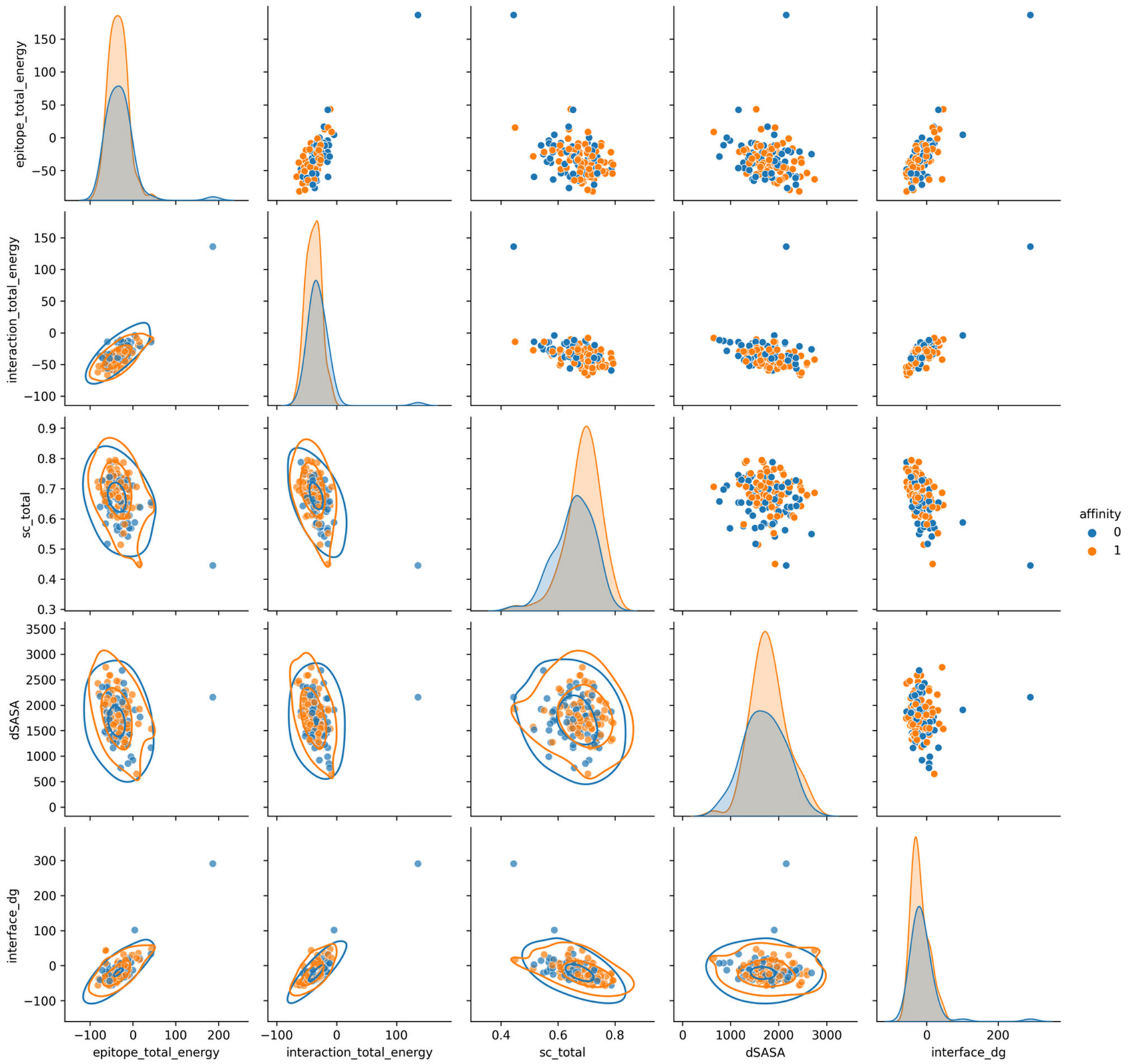

## 2.2 **Figure S2:** Pairwise association matrices for the top 5 features within the dMaSIF feature-set.

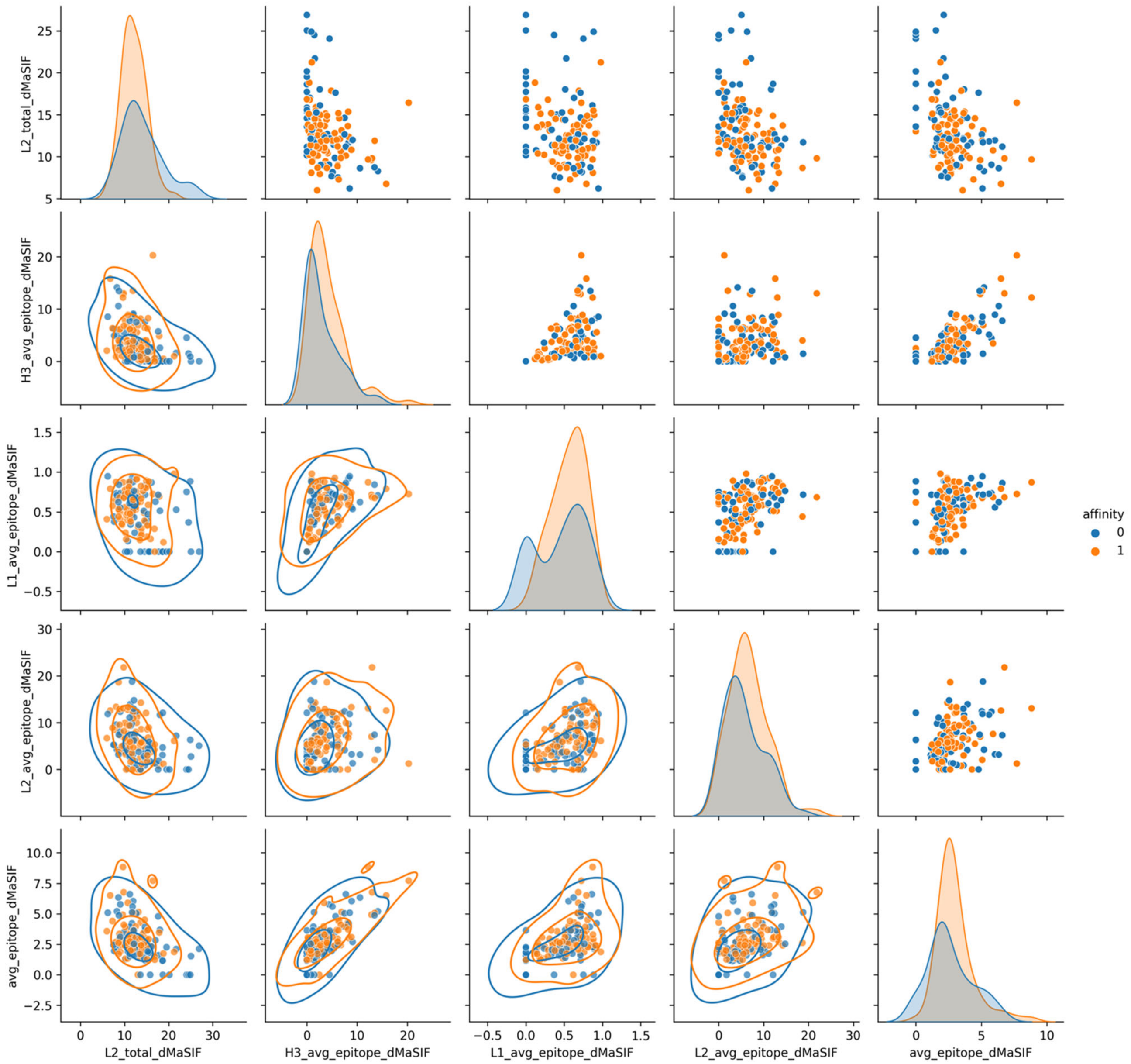

**2.3 Figure S3:** Pairwise association matrices for the top 5 features within the statistical (AIF) feature-set.

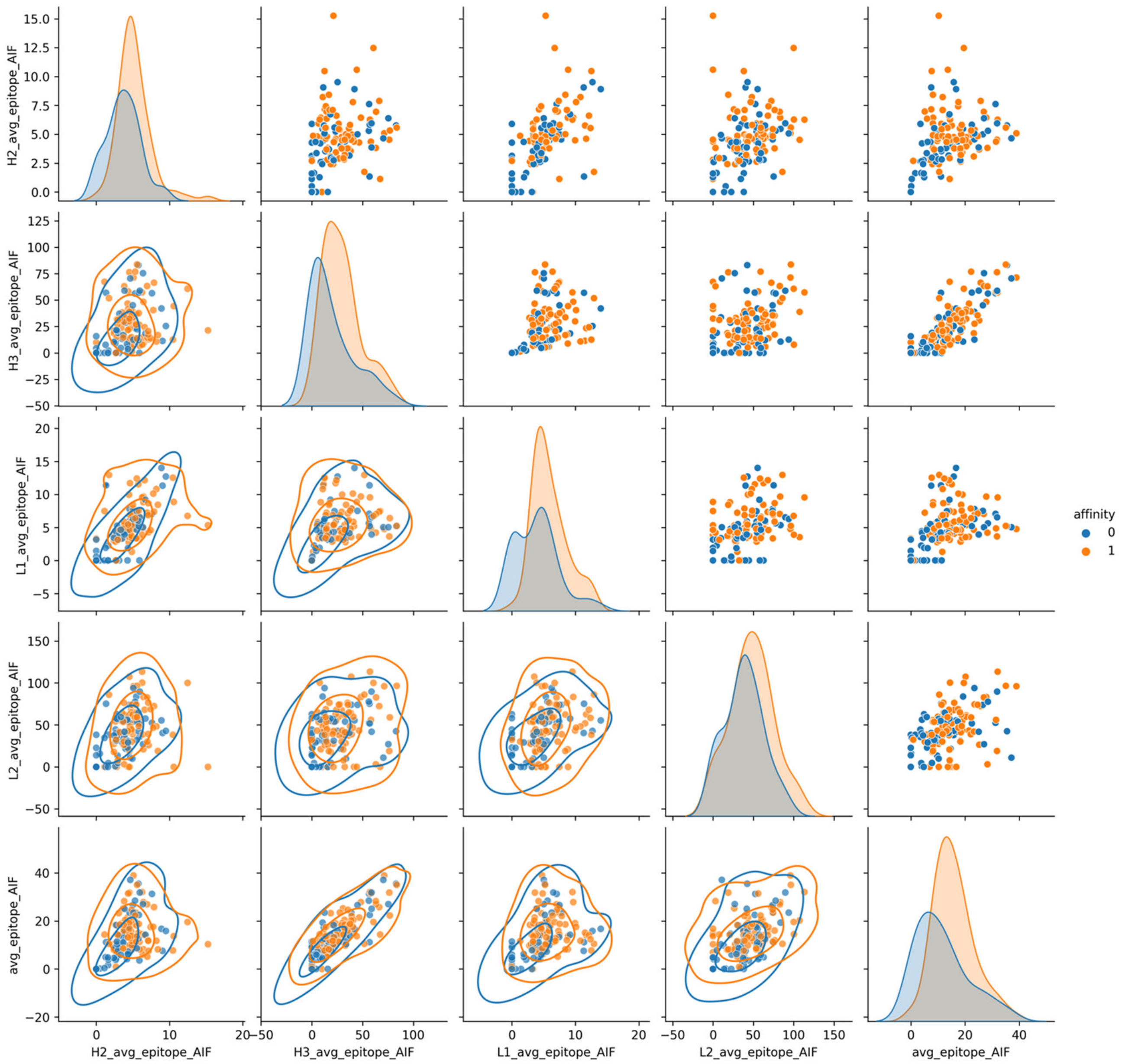

**2.4 Figure S4:** Pairwise association matrices for the top 5 features within the networking (SIN) feature-set.

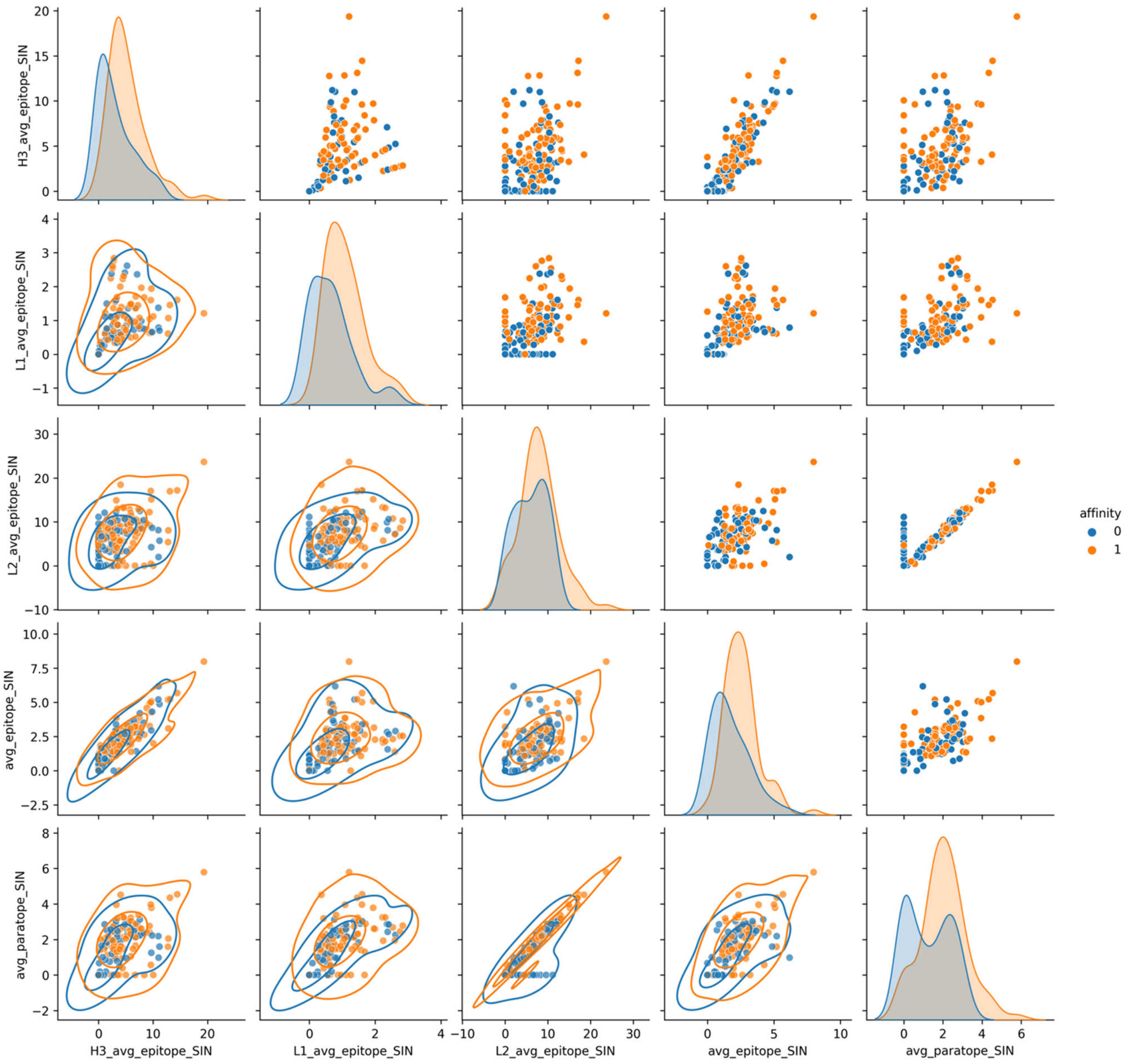

## 2.5 **Figure S5:** Pairwise association matrices for the top 5 features within the aa_counts feature-set.

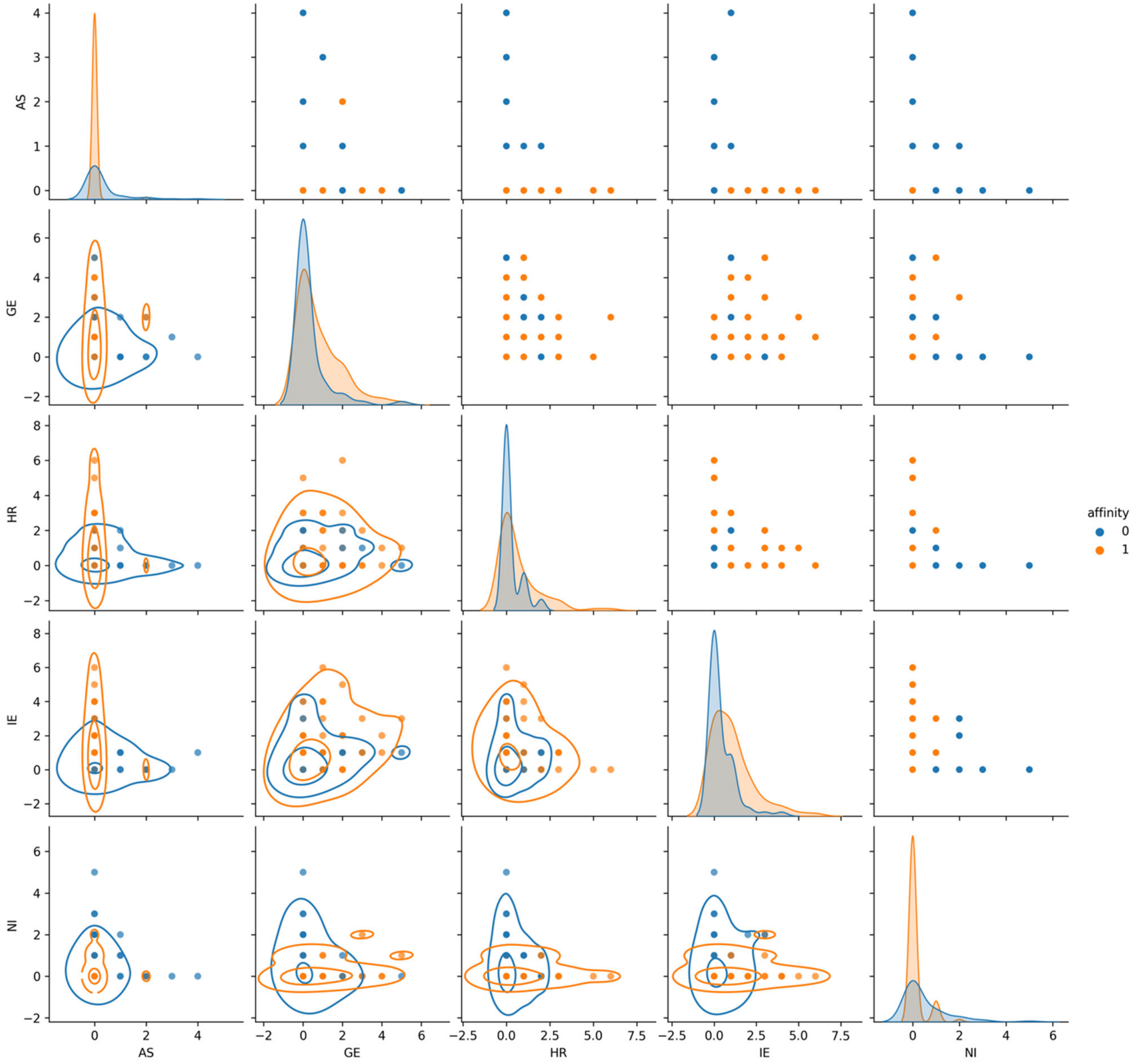

**2.6 Figure S6:** Pairwise association matrices for the top 5 features within the aa_counts_CDR feature-set.

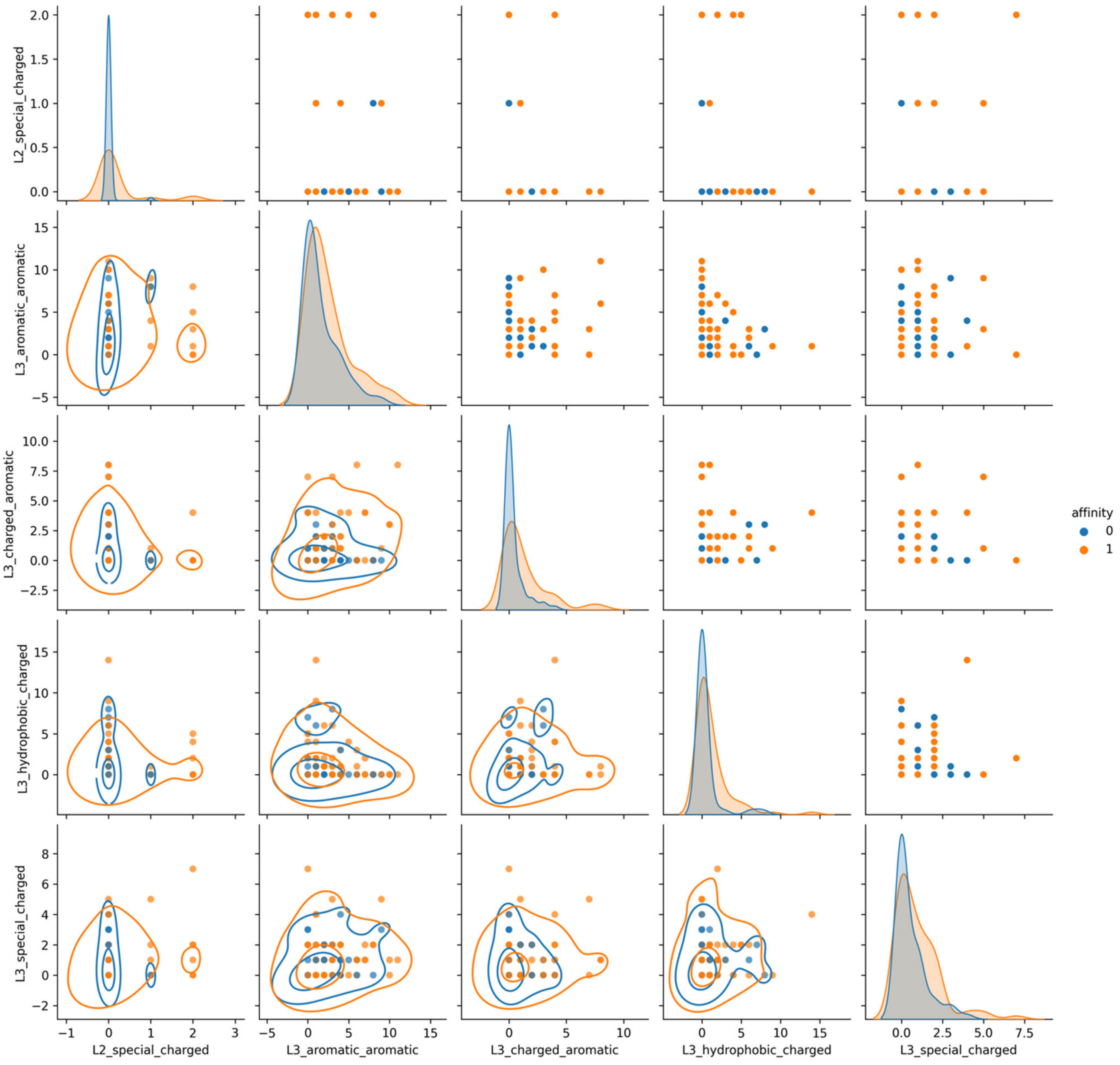

**2.7 Figure S7:** Pairwise association matrices for the top 5 features within the multivalent_interactions feature-set.

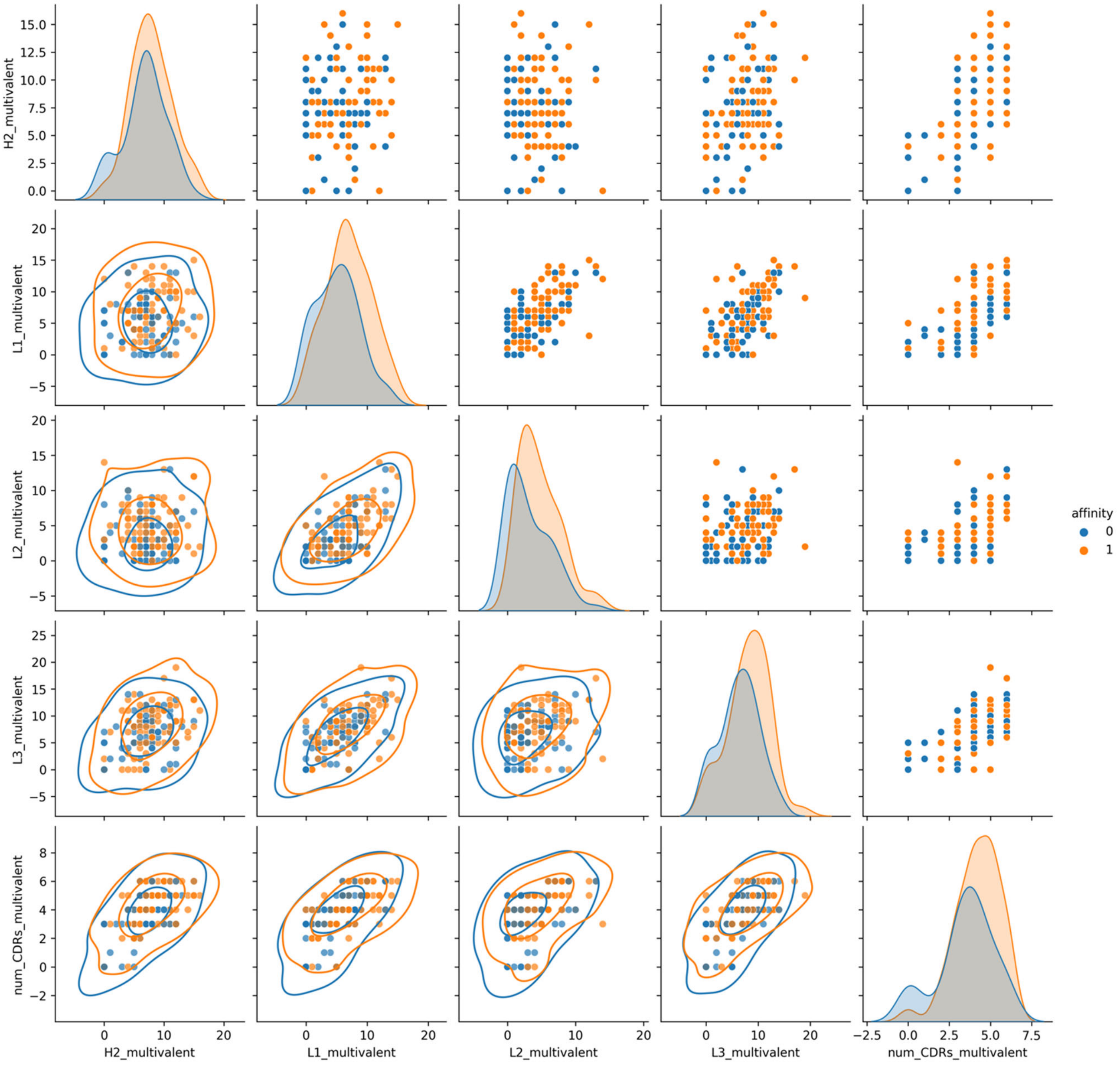

## 2.8 **Figure S8:** Pairwise association matrices for the top 5 features within the antibody_info feature-set.

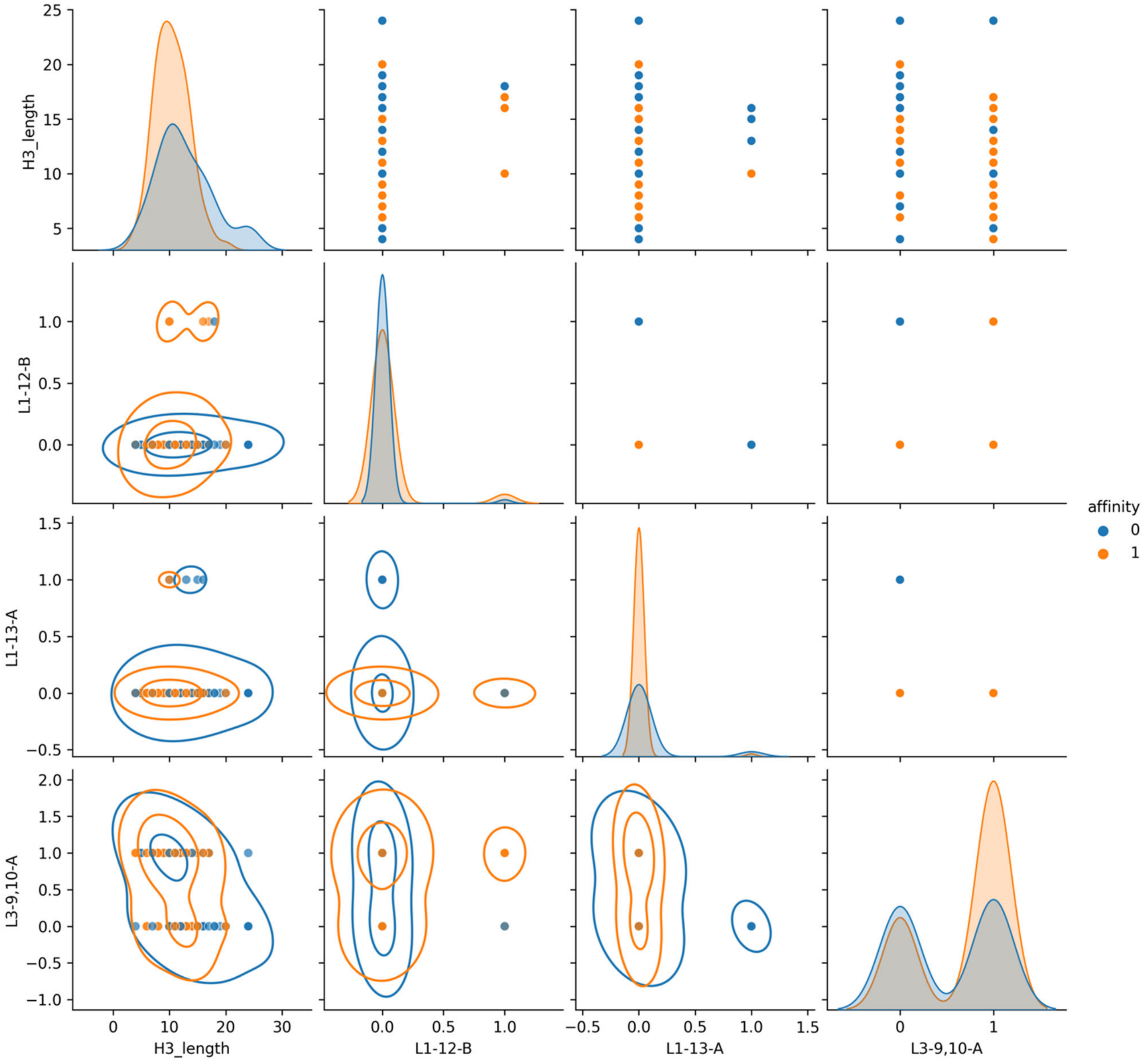

**2.9 Figure S9:** Antibody affinity versus CDR-H3 length for the entire dataset of 356 affinity-labeled antibodies.

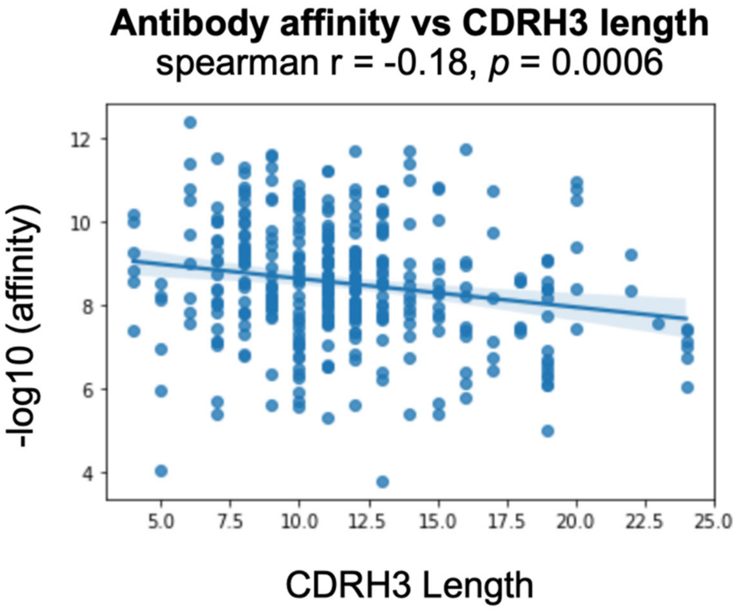

Supplement: Supplementary file 1 [file DataSheet1.PDF]
